# Sex hormone changes during weight loss and maintenance in overweight and obese postmenopausal African-American and non-African-American women

**DOI:** 10.1186/bcr3346

**Published:** 2012-10-31

**Authors:** Rachael Z Stolzenberg-Solomon, Roni T Falk, Frank Stanczyk, Robert N Hoover, Lawrence J Appel, Jamy D Ard, Bryan C Batch, Janelle Coughlin, Xu Han, Lillian F Lien, Christina M Pinkston, Laura P Svetkey, Hormuzd A Katki

**Affiliations:** 1 Division of Cancer Epidemiology and Genetics, National Cancer Institute, National Institutes of Health, Department of Health and Human Services, 6120 Executive Boulevard, Rockville, MD 20854, USA; 2 Reproductive Endocrine Research Laboratory, Livingston Research Building, 1321 N. Mission Road, Room 201, Los Angeles, CA 90033, USA; 3 Welch Center for Prevention, Epidemiology, and Clinical Research, Johns Hopkins Medical Institutions, 2024 East Monument Street, Room 2-642, Baltimore, MD 21287, USA; 4 Epidemiology and Prevention, Maya Angelou Center for Health Equity, Wake Forest University Baptist Medical Center, Medical Center Boulevard, Winston Salem, NC 27157, USA; 5 Division of Endocrinology, Metabolism and Nutrition, Duke University Medical Center, 200 Trent Drive, Duke South Orange Zone, DUMC Box 3031, Durham, NC 27710, USA; 6 Department of Psychiatry and Behavioral Sciences, Johns Hopkins School of Medicine, 600 N. Wolfe Street, Meyer 101, Baltimore MD 21287, USA; 7 Division of Endocrinology, Metabolism, and Nutrition, Sarah Stedman Nutrition and Metabolism Center, Duke University Medical Center, Box 2956 DUHS, 201 Trent Drive, Durham, NC 27710, USA; 8 Sarah W. Stedman Nutrition and Metabolism Center, Duke Hypertension Center, 3475 Erwin Road, Suite 101D, Durham, NC 27705, USA

## Abstract

**Introduction:**

Changes in sex hormones with weight loss might have implications for breast cancer prevention but have not been examined extensively, particularly in African-American (AA) women.

**Methods:**

We conducted a prospective study of 278 overweight/obese postmenopausal women (38% AA) not taking hormone therapy within the Weight Loss Maintenance Trial. All participants lost at least 4 kg after a 6-month weight-loss phase and attempted to maintain weight loss during the subsequent 12 months. We evaluated the percentage changes in estrone, estradiol, free estradiol, testosterone, free testosterone, androstenedione, dehydroepiandrosterone sulfate and sex hormone-binding globulin (SHBG) using generalized estimating equations.

**Results:**

In all study phases, AA women had higher levels of estrogen and testosterone concentrations, independent of adiposity. On average, participants lost 7.7 kg during the weight-loss phase, and concentrations of estrone (-5.7%, *P *= 0.006), estradiol (-9.9%, *P *<0.001), free estradiol (-13.4%, *P *<0.0001), and free testosterone (-9.9%, *P *<0.0001) decreased, while the SHBG concentration (16.2%, *P *<0.001) increased. Weight change did not significantly affect total testosterone or other androgen concentrations. Compared with non-AA women, AA women experienced less change in estrogens per kilogram of weight change (that is, per 1 kg weight loss: estrone, -0.6% vs. -1.2%, *P*-interaction = 0.10; estradiol, -1.1% vs. -1.9%, *P*-interaction = 0.04; SHBG, 0.9% vs. 1.6%, *P*-interaction = 0.006; free estradiol, -1.4% vs. -2.1%, *P*-interaction = 0.01).

**Conclusion:**

To the best of our knowledge this is the first study to examine and compare the effects of intentional weight loss and maintenance on a panel of sex hormones in AA women and non-AA women. Although speculative, these data suggest hormonal differences may contribute to different racial patterns of breast cancer incidence and mortality and encourage further investigations to understand the long-term effects of weight loss on sex hormones in obese postmenopausal women.

**Trial Registration:**

ClinicalTrials.gov: NCT00054925

## Introduction

Over the past several decades, the prevalence of obesity has increased in the United States [1]. Nearly 70% of postmenopausal women are overweight or obese [1]. These women experience a 30 to 50% greater risk of breast cancer compared with leaner women [2]. The International Agency for Research on Cancer estimates that 25% of breast cancer cases may be attributable to obesity and sedentary lifestyle [2]. These associations may be due to alterations in endogenous hormones, including bioavailable sex steroids [3].

Obesity is linked to higher levels of circulating sex steroid hormone concentrations in postmenopausal women not taking hormone replacement [4], but studies examining the long-term effects of intentional weight reduction on endogenous sex hormones are surprisingly limited. Most studies have been small with durations <6 months [5-19]. For the majority of studies, weight loss was the consequence of a low-fat diet or an exercise intervention [5-19]. To the best of our knowledge, only one recent adequately powered clinical trial has examined the direct effects of weight loss on sex hormones [20] and none have examined weight loss maintenance. Additionally, none of these studies have included a substantial number of African-American (AA) women, a population that has a greater prevalence of obesity [21], unique incidence patterns of hormone-receptor-defined breast cancer subtypes [22], and higher breast cancer mortality when compared with Caucasian women. A better understanding of the long-term effects of weight loss on endogenous sex hormones in obese postmenopausal women may provide clues to the racial differences in breast cancer incidence and prognosis, and might have major implications for prevention.

We conducted a study among postmenopausal women participants of the Weight Loss Maintenance Trial (WLM) [23] who were overweight or obese at study entry and not taking hormone replacement. The objective of our study was to determine the effects of weight loss and maintenance of weight loss on sex hormone concentrations, overall and by race (AA and non-AA).

## Materials and methods

### Weight Loss Maintenance Trial

A detailed description of the WLM is published elsewhere [23,24]. The WLM (clinicaltrials.gov NCT00054925) was a multicenter, randomized trial of 1,032 men and women with the primary aim to test the effects of two interventions compared with an advice-only control group for maintaining initial weight loss. The primary outcome of the trial was weight.

The trial had two phases. Phase 1 was a nonrandomized 6-month group-based intensive behavioral weight loss intervention of 1,685 participants led by a trained interventionist that promoted reducing caloric intake and increasing physical activity (to at least 180 minutes/week) to lose 1 to 2 pounds per week [23]. Participants were encouraged to follow the Dietary Approaches to Stop Hypertension diet, which is high in fiber and low in fat content [25]. Major inclusion criteria for participation in phase 1 were: body mass index (BMI) between 25 and 45 kg/m^2^; current medication use for hypertension, dyslipidemia, or both; access to a telephone and to the Internet; and ability to keep a food diary for 5 days during the screening. Major exclusion criteria were medication-treated diabetes mellitus, a recent cardiovascular event or other medical or psychiatric condition that would preclude full participation in the study, weight loss >9 kg during the last 3 months, recent use of weight loss medications and prior weight loss surgery or scheduled surgery for this purpose [24].

Those who lost at least 4 kg in phase 1 were eligible for phase 2 of the WLM, a three-arm trial in which 1,032 individuals were randomized into one of two weight-maintenance interventions or a minimal care, self-directed/usual care control group [23]. The two interventions were a Personal Contact intervention that provided monthly personal contacts by a trained interventionist primarily via telephone, or an Interactive Technology intervention that provided unlimited contacts through an interactive Web-based program supplemented by other communication technologies [23]. The duration of phase 2 was 30 months.

Recruitment for the 6-month intensive behavioral weight loss program, phase 1 occurred between August 2003 and July 2004 [23]. Those who completed phase 1 and qualified for phase 2 were randomized from February through December 2004 to one of the three intervention groups [23]. Data collection was completed in June 2007 [23].

The study was approved by an institutional review board at each of four participating clinical sites, by the study's coordinating center, by a protocol review committee appointed by the National Heart, Lung, and Blood Institute, and by the Office of Human Subjects Research at the National Institutes of Health. Participants provided an informed consent for phase 1 and phase 2.

### Data collection

For our analyses, we used data collected at three points: study entry; 6 months later at the end of phase 1, which was the point of randomization into phase 2; and 12 months after randomization [23]. Height was measured using a calibrated, wall-mounted stadiometer at entry. Weight was measured in duplicate according to the WLM protocol using a high-quality, calibrated digital scale by trained staff members who were masked to treatment assignment [23]. At the end of the weight loss intervention and 12 months later, weight was measured on two separate days and values were averaged. Diet was assessed using a self-administered Block Food Frequency Questionnaire (NutritionQuest, Berkeley, CA. USA). Physical activity was measured using a calibrated, triaxial accelerometer (RT3; Stayhealthy Inc, Monrovia, CA, USA) for ≥10 hoursper/day for at least 4 days, including one weekend day. Accelerometry measures were used to calculate weekly minutes of moderate to vigorous physical activity. Participants self-reported their race.

### Identification of postmenopausal women

We administered a questionnaire to all female participants during the weight loss program to determine menopausal status. Menopause was defined as no menstrual periods during the last 12 months and/or surgical removal of both ovaries. Women older than 60 years were considered postmenopausal. For women between the ages of 48 and 60 years we confirmed menopausal status by considering levels of follicular stimulating hormone and estradiol from blood samples. Hormone replacement use was determined from questions that ascertain medications during the scheduled data collection. Among the 654 female participants in the trial, we identified from the questionnaire 313 postmenopausal participants who were not taking hormone replacement therapy at any time during the trial. From these participants, we further excluded 35 women, including 29 with high levels of total estradiol (>50 pg/ml, an indication that they were taking estrogen therapy or were not menopausal) and six with low follicular stimulating hormone (<30 mIU/ml, suggesting not menopausal). In total, 278 postmenopausal women were included in our analyses.

### Collection of blood samples and laboratory analysis of sex hormones

We collected 2 ml serum samples from the study participants after an overnight fast at three time points: prior to the weight loss intervention (phase 1 entry), 6 months later (the end of phase 1 and randomization into phase 2), and at 12 months after randomization (also 18 months after entry). The blood samples were processed, then sent to and stored at -70°C at the WLM biorepository, and transferred to the National Cancer Institute biorepository prior to laboratory measures. The three serum samples collected from the same participant were analyzed consecutively as triplets within each batch to minimize the laboratory variation between samples from the same subject.

The stored serum samples were shipped on dry ice to Dr Frank Stanczyk's laboratory at University Southern California Keck School of Medicine, where estrone, estradiol, testosterone, androstenedione, dehydroepiandrosterone sulfate (DHEAS) and sex hormone-binding globulin (SHBG) were measured in 2008. Estrone, total estradiol, testosterone and androstenedione were measured by validated radioimmunoassays after organic solvent extraction and Celite column partition chromatography [26,27]. DHEAS and SHBG were measured by direct immunoassays using the Immulite analyzer (Diagnostic Products Corporation, Inglewood, CA, USA). Free estradiol and free testosterone concentrations were calculated from measured estradiol, total testosterone, and SHBG with albumin assumed to be a constant (40 g/l) using the method by Sodergard and colleagues according to the law of mass action [28]. The lowest concentration of detection for estradiol was 3 pg/ml. Estradiol values below the detection limits were assigned a value of 3 pg/ml. There were three women at baseline, six women after the weight loss intervention at randomization, and 10 women at the 12-month weight loss maintenance phase that had estradiol concentrations below the detection limits (values <3 pg/ml). We also included 10% blinded quality control samples (*n *= 94) from two pools of serum composed from thin and overweight/obese postmenopausal women not taking hormone replacement therapy. Duplicate blinded quality control samples were placed in each batch. Using a nested components of variance analysis, with logarithmically transformed quality control measurements across all batches [29], the estimated overall (intrabatch and interbatch) coefficients of variation for estrone, estradiol, testosterone, androstenedione, DHEAS, and SHBG were 20%, 4.3%, 10%, 10%, 8.4%, and 4.3%, respectively.

### Statistical analysis

We analyzed the effect of weight loss and weight loss maintenance on hormone concentration as a prospective cohort without regard to treatment, since all participants in our study lost at least 4 kg and we assumed that changes in hormone concentrations are mediated through weight regardless of randomization to the weight maintenance interventions. To investigate the effect of weight loss on the logarithm of each hormone, we fit separate marginal regression models using generalized estimating equations with an independence working correlation matrix to estimate robust standard errors that accounted for multiple measurements over time within a subject. These models estimated the mean, and the percentage change in, hormone concentrations over each study phase. All models were adjusted for race, age, enrollment BMI, physical activity, and phase. Other variables examined as potential confounders included education, smoking history, alcohol use, energy, total and saturated fat and carbohydrate intake, dyslipidemia medication use, history of oopherectomy, endogenous hormones, and SHBG.

**Figure 1:**
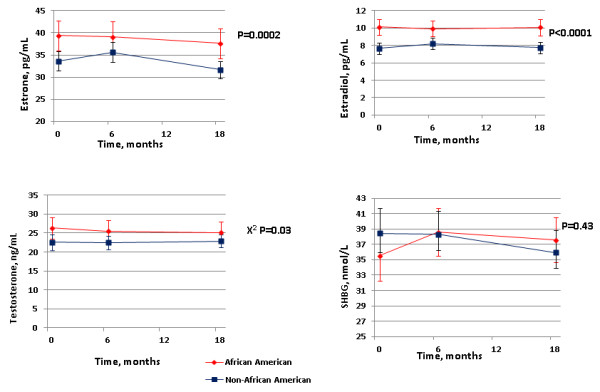
**Hormone concentrations by race in the Weight Loss Maintenance Trial, United States, 2003-2005**. Estrone, estradiol, testosterone, and sex hormone binding globulin (SHBG) concentration by race in the Weight Loss Maintenance Trial, United States, 2003-2005. Mean hormone concentrations during weight loss and weight maintenance within race groups, and 95% confidence intervals from generalized estimating equations adjusted for age, physical activity, phase, and body mass index (kg/m^2^) at each time (0, 6, and 18 months). *P *values from a chi-square test on three degrees of freedom.

We used generalized estimating equations models to test whether hormone levels differ by race, adjusted for BMI at each time (0, 6 and 18 months), using chi-square tests on three degrees of freedom (Figure 1). Modification of the effect of weight change by race, trial intervention, enrollment BMI, age, physical activity, and dyslipidemia medication were assessed by stratifying the models on levels of each factor and testing whether the weight change coefficients were equal with *t *tests. For the analysis of the modification of the effect of weight change by race and enrollment BMI, we additionally combined data over the study phase because the phase did not modify the effect of weight.

## Results

The mean age at study entry was 59.3 years (range 46 to 78 years), 37.8% were AA, 90% were postmenopausal for at least 2 years at the start of the trial, and 40% were taking dyslipidemia medication (Table 1). Compared with the non-AA women, the AA women were more likely to have a higher BMI and severe obesity (BMI >35 kg/m^2^), had a surgical menopause, had lower income, and presented less dyslipidemia medication use (Table 1).

**Table 1:** Baseline characteristics of postmenopausal women not taking hormones, Weight Loss Maintenance Trial

Characteristic	Overall (*n *= 278)	African-American (*n *= 105)	Non-African-American (*n *= 173)
Age (years)	46 to 78	47 to 73	46 to 78
Age	59.30 (6.32)	58.50 (6.08)	59.76 (6.43)
Race/ethnicity^a^			
Black/African-American	105 (37.77%)		
White/Caucasian	170 (61.15%)		
Native American	1 (0.36%)		
Asian	1 (0.36%)		
Body mass index (kg/m^2^)	33.4 (4.9)	34.3 (4.6)	32.9 (4.9)
World Health Organization category			
Overweight: 25 to 30 kg/m^2^	83 (29.9%)	22 (21.0%)	61(35.3%)
Obesity			
Stage 1: 30 to 34.9 kg/m^2^	98 (35.3%)	38 (36.2%)	60 (34.7%)
Stage 2: ≥35 kg/m^2^	97 (34.9%)	45 (42.9%)	52 (30.1%)
Physical activity (MVPA/week)	91.7 (86.1)	82.0 (69.9)	97.3 (93.9)
Alcohol (g)	4.3 (8.1)	2.1 (4.1)	5.6 (9.5)
Years since periods stopped^a^			
≤4 years	62 (22.30%)	20 (19.05%)	42 (24.28%)
5 to 9 years	39 (14.03%)	15 (14.29%)	24 (13.87%)
10 to 19 years	77 (27.70%)	31 (29.52%)	46 (26.59%)
20 years	46 (16.55%)	16 (15.24%)	30 (17.34%)
Reason periods stopped			
Natural menopause	163 (58.6%)	53(50.5%)	110 (63.6%)
Surgery (uterus and/or ovaries removed)	100 (36.0%)	48(45.7%)	52(30.1%)
Bilateral oopherectomy	51(18.3%)	20 (19.0%)	31 (17.9%)
Other	15 (5.4%)	4 (3.8%)	11(6.4%)
Age periods stopped (years)			
<45 years	83 (29.9%)	40 (38.1%)	43 (24.9%)
45 to 49 years	55 (19.8%)	21 (20.0%)	34 (19.7%)
50 to 52 years	56 (20.1%)	18 (17.1%)	38 (22.0%)
>53 years	81 (29.1%)	25 (23.8%)	56 (32.4%)
Age of menarche (years)^a^			
≤11 years	138 (49.6%)	50 (48.5%)	88 (50.9%)
12 to 13 years	83 (29.9%)	26 (24.8%)	57 (32.9%)
>14 years	54 (19.4%)	28 (26.7%)	26 (15.0%)
Education			
College degree	157 (56.48%)	58 (55.24%)	99 (57.23%)
Household income			
≥$60,000	111 (39.93%)	38 (36.19%)	73 (42.20%)
Smoker			
Never	160 (57.55%)	60 (57.14%)	100 (57.80%)
Former	105 (37.77%)	40 (38.10%)	65 (37.57%)
Current	13 (4.68%)	5 (4.76%)	8 (4.62%)
Dyslipidemia medication use	111 (39.93%)	31 (29.52%)	80 (46.24%)
Statin use	102 (36.69%)	29 (27.62%)	73 (42.20%)

Data presented as range, mean (standard deviation) or *n *(%). MVPA, moderate to vigorous physical activity. ^a^Proportions do not add to 100% due to missing data: years after menopause, *n *= 54; race, *n *= 1 refused; education, *n *= 3; household income, *n *= 20; age of menarche, *n *= 3.

Overall, participants averaged a 7.7 kg weight loss by the end of the weight loss phase (entry vs. 6 months), with significant reductions in estrone (-5.7%), estradiol (-9.9%), free estradiol (-13.4%), and free testosterone (-9.9%) concentrations, and an increase in SHBG (16.2%) concentrations (Table 2, columns 5 and 6). During the weight maintenance phase overall (6 months vs. 18 months), body weight increased 2.2 kg on average while estrone (-6.4%) and SHBG (-8.0%) decreased and free testosterone (5.0%) increased; otherwise, there were no other significant changes (Table 2, columns 7 and 8). The total testosterone and other androgens (androstenedione and DHEAS) did not significantly change, except that among the women with stage 2 obesity (BMI >35 kg/m^2^) testosterone decreased (-6.0%, *P *= 0.04) after the initial weight loss (average -8.3 kg) and increased (8.2%, *P *= 0.02) during the weight maintenance phase with an average weight gain of 2.3 kg. After the initial weight loss, those who gained weight had significant decreases in SHBG (-14.5%) and consequently increases in free estradiol (9.0%) and free testosterone (10.4%), but no change in other hormone concentrations (Table 3). Those who maintained their weight loss did not have significant changes in sex hormones except for a slight decrease in SHBG (-5.3%). Those who continued to lose weight had further significant reductions in the level of estrone (-5.2%) and increases in androstenedione (8.8%).

**Table 2:** Hormone change during weight loss and maintenance among postmenopausal women not taking hormones

	Months since study entry			Effect of weight on hormones			
	
	Study entry	End of weight loss phase	Weight loss maintenance	Weight loss, entry vs. 6 months		Weight maintenance, 6 months vs. 18 months	
	
	Initial	6 months	18 months	% Δ	*P *value	% Δ	*P *value
Weight (kg)^a^	89.1 (14.8)	81.4 (14.4)	83.6 (15.6)	-7.7	<0.0001	2.2	<0.0001
Hormone							
Estrone (pg/ml)	38.1 (1.1)	35.9 (1.0)	33.6 (1.0)	-5.7	0.006	-6.4	0.003
Estradiol (pg/ml)	9.4 (0.3)	8.4 (0.3)	8.5 (0.3)	-9.9	<0.0001	0.5	0.85
Testosterone (ng/ml)	24.6 (0.9)	23.7 (0.8)	24.0 (0.8)	-3.5	0.13	1.1	0.62
Androstenedione (pg/ml)	437 (12.1)	434 (11.9)	438 (12.9)	-0.5	0.76	0.8	0.68
Dehydroepiandrosterone sulfate (µg/dl)	47.0 (1.8)	47.8 (1.8)	46.6 (1.9)	1.7	0.31	-2.5	0.18
Sex hormone binding globulin (nmol/l)	35 (1.0)	40 (1.0)	37 (1.0)	16.2	<0.0001	-8.0	<0.0001
Free estradiol (pg/ml)	0.26 (0.01)	0.22 (0.01)	0.23 (0.01)	-13.4	<0.0001	3.7	0.18
Free testosterone (pg/ml)	5.3 (0.2)	4.8 (0.2)	5.1 (0.2)	-9.9	<0.0001	5.0	0.02

*n *= 278. Generalized estimating equations models used to estimate the mean (standard error) and percentage change (% Δ) in hormone concentrations over each study phase adjusted for age, physical activity, phase, initial body mass index (kg/m^2^), and race. ^a^Row represents the mean (standard deviation) for weight at the start of each phase, average weight change between each phase and overall.

**Table 3:** Hormone changes during weight maintenance phase, stratified by changes in weight criteria

	Months since study entry		Effect of weight change on hormones	
	
	End of weight loss phase	Weight loss maintenance	6 months vs. 18 months	
	
	6 months	18 months	% Δ	*P *value
**Weight gain >3% (*n *= 141)**				
Weight (kg)^a^	82.2 (13.8)	87.6 (14.77)	5.4	<0.0001
Hormone				
Estrone (pg/ml)	35.6 (1.6)	34.8 (1.5)	-2.2	0.50
Estradiol (pg/ml)	8.4 (0.4)	8.8 (0.4)	4.2	0.20
Testosterone (ng/dl)	21.5 (1.1)	22.1 (1.1)	2.7	0.36
Androstenedione (pg/ml)	417 (17.1)	406 (18.1)	-2.9	0.34
Dehydroepiandrosterone sulfate (µg/dl)	48.6 (2.8)	47.6 (2.9)	-2.0	0.34
Sex hormone binding globulin (nmol/l)	38.2 (1.3)	32.6 (1.1)	-14.5	<0.0001
Free estradiol (pg/ml)	0.22 (0.01)	0.24 (0.01)	9.0	0.006
Free testosterone (pg/ml)	4.5 (0.2)	4.9 (0.3)	10.4	0.0007
**Weight maintainers, ≤3% weight gain (*n *= 68)**				
Weight (kg)^a^	82.6 (15.2)	84.0 (15.5)	1.4	<0.0001
Hormone				
Estrone (pg/ml)	37.2 (1.9)	35.3 (1.9)	-5.2	0.17
Estradiol (pg/ml)	9.4 (0.7)	9.2 (0.6)	-2.3	0.55
Testosterone (ng/dl)	27.1 (1.9)	26.4 (1.7)	-2.6	0.59
Androstenedione (pg/ml)	473 (22.0)	475 (22.2)	0.4	0.92
Dehydroepiandrosterone sulfate (µg/dl)	50.1 (3.6)	47.3 (3.4)	-5.6	0.16
Sex hormone binding globulin (nmol/l)	39.1 (2.1)	37.0 (1.9)	-5.3	0.01
Free estradiol (pg/ml)	0.25 (0.02)	0.25 (0.02)	-1.1	0.78
Free testosterone (pg/ml)	5.6 (0.4)	5.6 (0.4)	-0.2	0.97
**Continued weight loss (*n *= 69)**				
Weight (kg)^a^	78.5 (14.6)	75.2 (14.0)	-3.4	<0.0001
Hormone				
Estrone (pg/ml)	37.0 (2.0)	31.4 (1.9)	-15.2	0.0003
Estradiol (pg/ml)	8.3 (0.6)	8.0 (0.7)	-3.8	0.60
Testosterone (ng/dl)	25.7 (1.6)	26.2(1.8)	2.1	0.55
Androstenedione (pg/ml)	434 (25.6)	472 (28.7)	8.8	0.02
Dehydroepiandrosterone sulfate (µg/dl)	44.4 (3.0)	44.4 (3.6)	-0.02	1.00
Sex hormone binding globulin (nmol/l)	47.4 (2.9)	48.3 (2.9)	1.9	0.48
Free estradiol (pg/ml)	0.21 (0.02)	0.2 (0.02)	-1.5	0.84
Free testosterone (pg/ml)	4.7 (0.3)	4.8 (0.3)	1.2	0.70

Generalized estimating equations models used to estimate mean (standard error) and percentage change (% Δ) in hormone concentrations over each study phase adjusted for race, age, physical activity, phase and initial body mass index (kg/m^2^). ^a^Row represents the mean (standard deviation) for weight at the start of each phase, average weight change between each phase and overall.

Combining data from both phases (Table 4), a 1 kg weight loss was associated with a 1.0% decrease and a 1.6% decrease in estrone and estradiol, respectively, a 1.3% increase in SHBG (*P *<0.0001), and consequently a 1.9% decrease and a 0.8% decrease in free estradiol and free testosterone, respectively, with no change in other androgen concentrations. However, the magnitude of these changes differed between AA women and non-AA women. Compared with non-AA women, AA women experienced less change in hormone per kilogram of weight lost, with estrone declining 0.6% versus 1.2% (*P*-interaction = 0.10), estradiol declining 1.1% versus 1.9% (*P*-interaction = 0.04), and free estradiol declining 1.4% versus 2.1% (*P*-interaction = 0.01), while SHBG increased less (0.9% vs. 1.6%, *P*-interaction = 0.006). Total testosterone and other androgen concentrations were unaffected by weight change for both races. On average, AA women experienced slightly less decrease in weight (-7.1 vs. -8.0 kg) and BMI (-2.7 vs. -3.0 kg/m^2^) after the intervention but a similar increase in weight (2.2 kg) and BMI (0.9 kg/m^2^) during weight maintenance. AA women also had higher circulating estrogen and testosterone concentrations compared with non-AA women (Figure 1), after controlling for BMI during all study phases.

**Table 4:** Change in hormone level per kilogram weight change over 18 months, overall and by race

Hormone	Overall (*n *= 278)		African-American (*n *= 105)		Non-African-American (*n *= 173)		*P*-interaction by race
		
	% Δ/kg	*P *value	% Δ/kg	*P *value	% Δ/kg	*P *value	
Estrone (pg/ml)	1.0	<0.0001	0.6	0.03	1.2	<0.0001	0.10
Estradiol (pg/ml)	1.6	<0.0001	1.1	<0.0001	1.9	<0.0001	0.04
Testosterone (ng/dl)	0.2	0.22	-0.02	0.95	0.3	0.15	0.30
Androstenedione (pg/ml)	-0.2	0.19	-0.2	0.44	-0.2	0.18	1.0
Dehydroepiandrosterone sulfate (μg/dl)	0.4	0.07	0.5	0.14	0.2	0.36	0.5
Sex hormone binding globulin (nmol/l)	-1.3	<0.0001	-0.9	<0.0001	-1.6	<0.0001	0.006
Free estradiol (pg/ml)	1.9	<0.0001	1.4	<0.0001	2.1	<0.0001	0.01
Free testosterone (pg/ml)	0.8	<0.0001	0.5	0.05	1.0	<0.0001	0.07

Generalized estimating equations models using the combined data across all phases of the logarithm of each hormone regressed on weight (kg) adjusted for race, age, physical activity, and phase. For example, overall estradiol increases 1.6% per kilogram of weight gain or decreases 1.6% per kilogram of weight loss. In African Americans, losing 1 kg means estradiol decreases 1.1% or gaining 1 kg means estradiol increases 1.1%; while in non-African Americans, losing or gaining 1 kg means estradiol decreases 1.9% or increases 1.9% respectively. The interaction or differences in percentage change (% Δ) in estradiol concentration by race is statistically significant (*P*-interaction = 0.04).

Exclusion of women who reported recent menopause and who might have higher estrogen concentrations (<4 years ago), have had bilateral oophorectomy (*n *= 51 women), or have nonmedicated diabetes did not substantially alter our results (data not shown). There were no significant interactions for the effect of weight change by enrollment BMI category, trial intervention (Tables S1, S2, S3 in Additional file 1), age, physical activity, or dyslipidemia medication use (data not shown).

## Discussion

To the best of our knowledge this is the first study to examine the consequence of intentional weight loss and weight loss maintenance on a panel of sex hormones in AA women and also compare the effects with those for non-AA women. In our study of overweight and obese postmenopausal women, intentional weight loss significantly reduced serum concentrations of estrone, estradiol, free estradiol and free testosterone, and increased SHBG, but had no effect on total testosterone and other androgen concentrations. The effect of weight loss differed by race, with AA women experiencing less change in estrone, estradiol, free estradiol, and SHBG per kilogram change than non-AA women. In addition, AA women had higher estrogen and testosterone concentrations, independent of adiposity. Sustaining the weight loss for the subsequent 12 months did not substantially change sex hormone concentrations except for slight increases in SHBG. For those who continued to lose weight during the weight maintenance phase, estrone continued to decrease.

One aspect of our study is that 38% of our population was AA. The AA women have higher mortality rates than non-AA women despite a lower overall incidence of breast cancer [22], and present with more aggressive disease distinguished by higher grade, poorer survival, and estrogen receptor (ER)-negative status [22,30]. Compared with non-AA women, the AA women in our study had higher concentrations of estrone, estradiol, and testosterone that were independent of BMI across all study phases and an attenuated change in estrogen concentrations with weight loss. These observations are consistent with the few recent cross-sectional studies demonstrating for AA women that circulating estrogens are higher than for non-AA women in both premenopausal and postmenopausal women [31-34].

While the ovaries are the primary source of estrogens in premenopausal women, estrogen synthesis also occurs in the adipose tissue [35]. Obesity and/or adult weight gain increase postmenopausal breast cancer risk in Caucasian women, particularly for ER-positive disease. The evidence for AA women, however, is equivocal [36], with positive, null, and inverse associations reported [30,36,37]. The reasons for these differences are unclear. Although speculative, the higher absolute levels and smaller change in estrogen concentrations in response to weight loss that we observe among AA women may provide a clue. There is experimental evidence that high levels of circulating estrogens promote the formation and progression of ER-negative breast tumors through a systemic increase in angiogenesis rather than by binding to the hormone receptor [35]. ER-negative breast cancer may also evolve from estrogen-responsive progenitor cells that lose their sensitivity to estrogens later in the carcinogenesis process [38].

Moreover, mammary stem cells have been shown to respond to sex hormone signaling, despite having an ER-negative phenotype [39]. This concept is indirectly supported epidemiologically by the transient increased incidence of breast cancer, particularly ER-negative tumors, that occurs after a full-term pregnancy, when estrogen concentrations are at their highest [35]. Further, parity is associated with higher rates of triple-negative disease in both Caucasian and AA women [40,41]. It is plausible that AA women have inherently higher estrogen concentrations compared with Caucasians, which contributes to the distinctive negative receptor phenotype of breast cancer in AA women that is independent of adiposity. The endogenous sex hormone differences could also contribute to the greater bone density observed among AA women [42,43], or, more broadly, the higher incidence of other hormone-related cancers among AA people [44]. A genetic component, such as genes related to steroid hormone metabolism, may perhaps explain the nature of breast tumor biology in women of African descent more strongly than adiposity [22].

Our results are consistent with a recent 12-month randomized controlled trial of postmenopausal women (the Nutrition and Exercise for Women trial) that examined the effects of caloric restriction, exercise, or both on sex hormone concentrations compared with those of a control group [20]. Both studies showed that weight loss as a result of caloric restriction and exercise significantly decreases estrogens and free testosterone, and increases SHBG with less striking reductions in total testosterone [20] and no significant changes in the other androgens [20]. Although exercise trials in sedentary, overweight or obese postmenopausal women have not shown consistent overall effects of exercise on sex hormone concentrations, they do suggest reductions in estradiol [15,45], testosterone [46,47], and androstenedione [47], and increases in SHBG [15], among women who experienced concomitant weight loss [15] and/or changes in body composition, specifically fat distribution [45-47]. Compared with these previous trials, our study had slightly more obese participants, none of whom had a normal weight prior to enrollment, and a higher proportion of AA women. In addition, our study was of longer duration with the goal to maintain lost weight for the subsequent year. The weight loss achieved by the WLM participants during the first 6 months was comparable with that of the caloric-restricted Nutrition and Exercise for Women trial participants, with and without physical activity, and substantially more than that of participants in the exercise trials. Higher compared with lower blood estrogen and testosterone concentrations have been associated with breast cancer risk in multiple prospective studies [48-53]. The Endogenous Hormone and Breast Cancer Collaborative Group showed in a pooled analysis that the significant positive association between BMI and postmenopausal breast cancer was reduced after adjustment for estrogens, particularly bioavailable estradiol, and SHBG, whereas adjustment for the androgens did not substantially change the BMI association [4]. Our results and those of others support the hypothesis that intentional weight loss might reduce the risk of breast cancer by decreasing estrogens more so than androgens [4,20].

Our findings are consistent with the known inter-relationship of obesity, physical activity, insulin, sex hormones and SHBG [54-56]. The decreases in estrogens we observed in overweight and obese postmenopausal women with sustained weight loss may be explained by declines in intra-abdominal body fat, which is the predominant site for estrogen production in postmenopausal women [54,55]. Weight loss also improves insulin sensitivity and the resulting lowered insulin levels enhance hepatic synthesis of SHBG [54], the protein that effectively binds both estradiol and testosterone, thereby limiting the amount of bioavailable steroid hormone in circulation [54]. Estrone is the predominant hormone present in postmenopausal women and is synthesized from androstenedione via aromatase in adipose tissue. The significant decrease in estrone concentrations among the women who continued to lose weight during the maintenance phase may possibly be in response to the sustained loss of adipose tissue. The lack of a significant change in SHBG within this group might be explained by a threshold effect related to maintaining the improved insulin sensitivity and lower insulin levels that occurred after the original weight loss.

The strengths of our study are the prospective design, the relatively large sample size with repeated measures of hormones on the same women over 18 months, and the valid and reliable measures of estrogens and androgens. We were also able to evaluate the effects on hormones of initial weight loss, as well as weight loss maintenance, weight regain, and continued weight loss. We avoid hormone replacement masking changes in endogenous sex hormones related to weight change because none of the women in our study were taking hormone replacement. Weight was measured in a standard manner by trained staff [23].

There are also limitations to the study. Our study only included overweight or obese women who successfully lost weight. The differences we observed in hormone concentrations between AA women and non-AA women might be explained by the slightly higher BMI of AA women compared with non-AA women in our study (Table 2); however, the fact that there was no significant interaction of the hormone changes by enrollment BMI category in our study (Tables S1, S2 in Additional file 1) would oppose this assertion. Although there was only a small number of women with estradiol concentrations below the detection limits, this could possibly influence our estradiol results towards the null because the true estradiol values are less than the values assigned. This influence could explain why estrone continued to significantly decrease with continued weight loss during the maintenance phase while estradiol did not. Current medication use for dyslipidemia was an inclusion criterion to participate in the WLM study. While it is possible that use of these medications could influence our results, they did not confound or modify the changes in hormone concentrations that we observed. We do not have information on family history of breast cancer, parity, or measures of other hormones such as insulin or leptin that might influence endogenous sex hormone concentrations. The racial differences in SHBG response to weight change might in part be explained by AA women having higher insulin concentrations. Studies of longer duration are needed to evaluate hormone changes associated with substantial weight regain and cycling that occur frequently among women. Finally, our study was not able to relate weight loss to cancer outcomes.

## Conclusion

Our results show that decreases in endogenous estrogens persist following intentional weight loss that is maintained for at least 12 months but the magnitude differs for AA women and non-AA women. Elevated levels of sex steroid hormones have been proposed to mediate many of the risk factors for postmenopausal breast cancer, including obesity, particularly for hormone receptor-positive tumors. As the prevalence of obesity among postmenopausal women increases worldwide, intervention efforts to promote and maintain weight loss may have a major public health impact for decreasing the burden of breast cancer. The racial hormone differences that we observe could have relevance to the distinctive tumor phenotype and biology of breast cancer in AA women, and possibly other hormone-related cancers in AA women, which deserves further investigation.

## Abbreviations

AA: African-American; BMI: body mass index; DHEAS: dehydroepiandrosterone sulfate; ER: estrogen receptor; SHBG: sex hormone-binding globulin; WLM: Weight Loss Maintenance Trial.

## Competing interests

The authors declare that they have no competing interests.

## Authors' contributions

RZS-S contributed to the concept of the study. RZS-S, RTF, and HAK contributed to the study design, analysis, and interpretation of the results. RZS-S and the Weight Loss Maintenance Collaborative Research Group (LJA, JDA, LPS) contributed to the acquisition of the data. RZS-S, RTF, HAK, CP, and XH contributed to the statistical analysis. RZS-S contributed to the funding of the ancillary study of postmenopausal women, and LJA and LPS to the WLM. FS measured the sex hormones. RZS-S drafted and revised the manuscript. RTF, FS, RNH, LJA, JDA, BCB, JC, XH, LFL, CP, LPS, and HAK provided substantive interpretation and editorial comments on manuscript drafts. All authors reviewed and approved the final manuscript.

## Supplementary Material

Additional file 1 **Table S1 presenting sex hormone changes during weight loss and weight loss maintenance within study entry BMI category**. Table S2 presenting the percentage change (% Δ) in hormone concentration per kilogram of weight change: overall and by BMI at study entry. Table S3 presenting weight and sex hormone changes during weight loss and weight loss maintenance by weight loss maintenance intervention. Click here for file
