# Supplementary material for: Sex hormone changes during weight loss and maintenance in overweight and obese postmenopausal African-American and non-African-American women
Source: Breast Cancer Res. 2012 Oct 31;14(5):R141. doi: 10.1186/bcr3346 (PMC3635052; doi:10.1186/bcr3346)
Supplement: Additional file 1 — Table S1 presenting sex hormone changes during weight loss and weight loss maintenance within study entry BMI category. Table S2 presenting the percentage change (% Δ) in hormone concentration per kilogram of weight change: overall and by BMI at study entry. Table S3 presenting weight and sex hormone changes during weight loss and weight loss maintenance by weight loss maintenance intervention. [file bcr3346-S1.DOC]

| Table s1. Sex hormone change during weight loss and weight loss maintenance within study entry Body Mass Index category a | | | | | | | | | |  | |
| --- | --- | --- | --- | --- | --- | --- | --- | --- | --- | --- | --- |
|  | Months Since Start of Trial | | | Effect of weight loss on hormones | | | | | | |  |
|  | Study entry | End of weight loss phase | Weight loss maintenance | Entry vs. 6 months | | | | 6 vs. 18 months | | | |
|  | Initial | 6 months | 18 months |  | |  | | | | |  |
|  | Mean (SE) | Mean (SE) | Mean (SE) | % Δ | p-value | | % Δ | | p-value | |  |
| ***Overweight, 25-29.9 kg/m2 (n = 83)*** |  |  |  |  |  | |  | |  | |  |
| Weight, kg (SD) b | 75.0 (6.3) | 68.2 (6.6) | 69.5 (7.7) | -6.8 | <.0001 | | 1.3 | | 0.0002 | |  |
| ***Hormone*** |  |  |  |  |  | |  | |  | |  |
| Estrone, pg/mL | 32.5 (1.9) | 30.8 (1.7) | 28.0 (1.6) | -5.2 | 0.26 | | -9.2 | | 0.04 | |  |
| Estradiol, pg/mL | 7.2 (0.4) | 6.7 (0.4) | 6.7 (0.4) | -6.9 | 0.10 | | 0.6 | | 0.92 | |  |
| Testosterone, ng/dL | 23.5 (2.0) | 22.1 (1.5) | 21.9 (1.6) | -5.6 | 0.30 | | -1.1 | | 0.71 | |  |
| Androstenedione, pg/mL | 406 (21) | 426 (22.7) | 434 (21.6) | 4.9 | 0.29 | | 2.1 | | 0.61 | |  |
| Dehydroepiandrosterone sulfate, ng/dL | 42.9 (3.4) | 44.2 (3.3) | 42.5 (3.3) | 3.0 | 0.33 | | -3.9 | | 0.17 | |  |
| Sex hormone binding globulin, nmol/L | 41.2 (2.4) | 47.3 (2.4) | 44.3 (2.3) | 14.9 | <.0001 | | -6.4 | | 0.0017 | |  |
|  |  |  |  |  |  | |  | |  | |  |
| ***Obesity, Stage 1, 30-34.9 kg/m2 (n = 98)*** |  |  |  |  |  | |  | |  | |  |
| Weight, kg(SD) b | 86.1 (7.9) | 78.3 (7.8) | 81.3 (9.6) | -7.8 | <.0001 | | 3.0 | | <.0001 | |  |
| ***Hormone*** |  |  |  |  |  | |  | |  | |  |
| Estrone, pg/mL | 37.0 (1.5) | 34.9 (1.5) | 33.5 (1.6) | -5.5 | 0.07 | | -4.2 | | 0.22 | |  |
| Estradiol, pg/mL | 9.1 (0.5) | 8.0 (0.4) | 8.3 (0.5) | -11.4 | 0.0037 | | 2.8 | | 0.54 | |  |
| Testosterone, ng/dL | 24.9 (1.4) | 25.1 (1.5) | 24.1 (1.5) | 0.9 | 0.81 | | -4.1 | | 0.29 | |  |
| Androstenedione, pg/mL | 485 (21.3) | 457 (19.2) | 443 (21.8) | -5.8 | 0.08 | | -3.1 | | 0.34 | |  |
| Dehydroepiandrosterone sulfate, ng/dL | 48.5 (3.1) | 49.0 (3.1) | 48.0 (3.3) | 0.8 | 0.78 | | -1.9 | | 0.61 | |  |
| Sex hormone binding globulin, nmol/L | 34.4 (1.7) | 41.8 (1.8) | 37.0 (1.8) | 21.5 | <.0001 | | -11.5 | | <.0001 | |  |
|  |  |  |  |  |  | |  | |  | |  |
| ***Obesity, Stage 2, >35 kg/m2 (n = 97)*** |  |  |  |  |  | |  | |  | |  |
| Weight, kg(SD) b | 104.1 (11.3) | 95.83 (11.3) | 98.1 (12.9) | -8.3 | <.0001 | | 2.3 | | 0.0001 | |  |
| ***Hormone*** |  |  |  |  |  | |  | |  | |  |
| Estrone, pg/mL | 44.7 (2.1) | 42.2 (2.0) | 39.5 (1.8) | -5.6 | 0.09 | | -6.5 | | 0.05 | |  |
| Estradiol, pg/mL | 12.6 (0.7) | 11.2 (0.6) | 11.0 (0.6) | -11.0 | 0.0021 | | -2.4 | | 0.53 | |  |
| Testosterone, ng/dL | 25.2 (1.3) | 23.6 (1.3) | 25.6 (1.5) | -6.0 | 0.04 | | 8.2 | | 0.02 | |  |
| Androstenedione, pg/mL | 413 (18.1) | 415 (18.9) | 431 (21.8) | 0.5 | 0.87 | | 3.8 | | 0.26 | |  |
| Dehydroepiandrosterone sulfate, ng/dL | 48.2 (3.2) | 49.1 (3.8) | 48.0 (3.3) | 2.0 | 0.41 | | -2.4 | | 0.41 | |  |
| Sex hormone binding globulin, nmol/L | 29.8 (1.3) | 33.3 (1.3) | 31.5 (1.2) | 11.6 | <.0001 | | -5.3 | | 0.02 | |  |
|  |  |  |  |  |  | |  | |  | |  |

a Generalized estimating equations (GEE) models used to estimate mean (standard error), and percent change in hormone concentrations over each study phase adjusted for, age, physical activity, phase and initial BMI (kg/m2)

b Weight, kg row represents mean (Standard Deviation, SD) for weight at start of each phase, average weight change between each phase and overall

| Table s2. Percent Change (% Δ) in hormone Concentration per kilogram weight change: overall and by body mass index at study entry a | | | | | | | | |  |  |
| --- | --- | --- | --- | --- | --- | --- | --- | --- | --- | --- |
|  | **Overweight** | | **Obese, Stage 1** | | **Obese, Stage 2** | |  |  | | |
|  | N=83 | | N=98 | | N=97 | |  |  | | |
|  | % Δ/kg | p-value | % Δ/kg | p-value | % Δ/kg | p-value | P-interaction |  | | |
| ***Hormone*** |  |  |  |  |  |  |  |  | | |
| Estrone, pg/mL | 0.9 | 0.18 | 1.2 | 0.001 | 0.7 | 0.03 | 0.9 |  | | |
| Estradiol, pg/mL | 1.6 | 0.04 | 1.3 | 0.01 | 1.3 | <0.0001 | 0.9 |  | | |
| Testosterone, ng/dL | -0.7 | 0.38 | 0.3 | 0.48 | 0.4 | 0.22 | 0.3 |  | | |
| Androstenedione, pg/mL | -0.7 | 0.23 | 0.04 | 0.91 | -0.1 | 0.76 | 0.3 |  | | |
| Dehydroepiandrosterone sulfate, ug/dL | 0.3 | 0.57 | 0.5 | 0.23 | 0.5 | 0.16 | 0.5 |  | | |
| Sex hormone binding globulin, nmol/L | -1.2 | 0.01 | -1.8 | <0.0001 | -1.2 | <0.0001 | 0.9 |  | | |

a Generalized estimating equations (GEE) models of the logarithm of each hormone regressed on weight (kg) adjusted for race, age, physical activity, and phase.

| Table s3. Weight and sex hormone change during weight loss and weight loss maintenance by weight loss maintenance intervention a | | | | | | | |
| --- | --- | --- | --- | --- | --- | --- | --- |
|  | Months since study entry | | | Effect of weight loss on hormones | | | |
|  | Study entry | End of weight loss phase | Weight loss maintenance |  | | | |
|  | Initial | 6 months | 18 months | Baseline vs. 6 months | | 6 vs. 18 months | |
|  | Mean (SE) | Mean (SE) | Mean (SE) | % Δ | p-value | % Δ | p-value |
| ***Self-directed (n = 91)*** |  |  |  |  |  |  |  |
| Weight, kg b | 88.8 (13.1) | 81.2 (13.1) | 84.9 (14.8) | -7.6 | <.0001 | 3.7 | <.0001 |
| ***Hormone*** |  |  |  |  |  |  |  |
| Estrone, pg/mL | 38.2 (1.8) | 37.0 (1.8) | 35.3 (1.7) | -3.0 | 0.48 | -4.7 | 0.27 |
| Estradiol, pg/mL | 10.0 (0.5) | 8.8 (0.5) | 9.3 (0.5) | -11.9 | 0.004 | 5.7 | 0.31 |
| Testosterone, ng/dL | 22.5 (1.1) | 22.3 (1.1) | 22.8 (1.2) | -0.6 | 0.82 | 1.9 | 0.55 |
| Androstenedione, pg/mL | 421.9 (22.8) | 426.8 (22.2) | 422.4 (23.6) | 1.2 | 0.76 | -1.1 | 0.77 |
| Dehydroepiandrosterone sulfate, ng/dL | 49.2 (3.4) | 49.6 (3.2) | 48.9 (3.2) | 0.7 | 0.80 | -1.4 | 0.60 |
| Sex hormone binding globulin, nmol/L | 34.3 (1.8) | 39.5 (2.0) | 36.1 (1.8) | 15.3 | <0.0001 | -8.7 | 0.0002 |
|  |  |  |  |  |  |  |  |
| ***Interactive Technology (n = 89)*** |  |  |  |  |  |  |  |
| Weight, kg b | 90.5 (15.6) | 82.8 (14.8) | 84.5 (15.2) | -7.7 | <.0001 | 1.7 | 0.0002 |
| ***Hormone*** |  |  |  |  |  |  |  |
| Estrone, pg/mL | 38.2 (2.0) | 36.9 (1.8) | 34.8 (1.8) | -3.5 | 0.33 | -5.8 | 0.11 |
| Estradiol, pg/mL | 9.7 (0.6) | 8.78 (0.5) | 8.72 (0.6) | -9.7 | 0.02 | -0.6 | 0.90 |
| Testosterone, ng/dL | 24.1 (1.3) | 24.5 (1.3) | 24.7 (1.4) | 1.7 | 0.50 | 0.9 | 0.79 |
| Androstenedione, pg/mL | 452.7 (18.9) | 461.0 (17.2) | 485.3 (19.5) | 1.8 | 0.57 | 5.3 | 0.13 |
| Dehydroepiandrosterone sulfate, ng/dL | 48.7 (3.3) | 51.2 (3.3) | 50.3 (3.6) | 5.2 | 0.06 | -1.9 | 0.60 |
| Sex hormone binding globulin, nmol/L | 35.1 (1.8) | 40.8 (1.9) | 37.9 (1.86) | 16.2 | <0.0001 | -7.1 | 0.001 |
|  |  |  |  |  |  |  |  |
| ***Personal Contact (n = 98)*** |  |  |  |  |  |  |  |
| Weight, kg b | 88.0 (15.7) | 80.3 (15.2) | 81.8 (16.5) | -7.7 | <.0001 | 1.4 | 0.004 |
| ***Hormone*** |  |  |  |  |  |  |  |
| Estrone, pg/mL | 38.2 (1.9) | 34.9 (1.8) | 32.1 (1.7) | -8.6 | 0.004 | -7.9 | 0.02 |
| Estradiol, pg/mL | 8.8 (0.6) | 8.2 (0.5) | 8.0 (0.5) | -6.4 | 0.08 | -2.6 | 0.51 |
| Testosterone, ng/dL | 27.4 (2.0) | 24.7 (1.8) | 24.9 (1.7) | -9.6 | 0.07 | 0.7 | 0.88 |
| Androstenedione, pg/mL | 432.9 (20.2) | 414.0 (21.2) | 409.0 (21.5) | -4.4 | 0.24 | -1.2 | 0.72 |
| Dehydroepiandrosterone sulfate, ng/dL | 43.2 (2.7) | 42.9 (2.8) | 41.5 (3.0) | -0.7 | 0.81 | -3.1 | 0.38 |
| Sex hormone binding globulin, nmol/L | 35.3 (1.6) | 41.0 (1.7) | 37.2 (1.7) | 16.0 | <0.0001 | -9.2 | <0.0001 |

a Generalized estimating equations (GEE) models used to estimate mean (standard error), and percent change in hormone concentrations over each study phase adjusted for, age, physical activity, phase and initial BMI (kg/m2)

b Weight, kg row represents mean (Standard Deviation, SD) for weight at start of each phase, average weight change between each phase and overall
